# Splenic marginal zone lymphoma associated with chronic inflammatory demyelinating polyradiculoneuropathy: a case report

**DOI:** 10.25122/jml-2025-0023

**Published:** 2025-07

**Authors:** Tarek Mohammed, Halimah Saleh, Huda Albraim, Sariya Khan, Sahar Albraim, Deema Bakhashab

**Affiliations:** 1 Department of Hematology, Saudi German Hospital, Jeddah, Saudi Arabia; 2 Department of Internal Medicine, Saudi German Hospital, Jeddah, Saudi Arabia; 3 General Practice, My Clinic, Jeddah, Saudi Arabia; 4 General Medicine Practice Program, Batterjee Medical College, Jeddah, Saudi Arabia; 5 Department of Internal Medicine, Soliman Fakeeh Hospital, Jeddah, Saudi Arabia

**Keywords:** splenic marginal zone lymphoma, SMZL, chronic inflammatory demyelinating polyradiculoneuropathy, CIDP, case report

## Abstract

Chronic inflammatory demyelinating polyradiculopathy (CIDP) is an acquired immune-mediated neuropathy characterized by progressive or relapsing-remitting proximal and distal weakness. Lymphomas are among various hematological malignancies associated with CIDP. Splenic marginal zone lymphoma (SMZL) is a rare, indolent B-cell non-Hodgkin lymphoma that classically presents with splenomegaly and cytopenia. The co-occurrence of SMZL and CIDP is extremely rare; the diagnosis thus presents a unique challenge both diagnostically and therapeutically. We report a 65-year-old male patient with progressive proximal weakness, night sweats, and splenomegaly. Investigations revealed pancytopenia with imaging studies confirming the splenomegaly. Further investigations, such as a bone marrow biopsy and histopathology of the spleen, were indicative of a hypocellular marrow and SMZL, respectively. Simultaneously, a diagnosis of CIDP was established based on clinical findings, as well as electromyography and nerve conduction studies. For CIDP, immunosuppressive therapy was initiated; however, no treatment was administered for SMZL due to its indolent nature. He showed partial neurological improvement with the treatment given for CIDP. This underlines the importance of treating both diseases. The rare association of CIDP and SMZL is presented in this case, highlighting the importance of a comprehensive diagnostic workup in patients with neurological and hematological abnormalities. Therefore, for the management of such patients, timely identification and appropriate therapeutic approaches will be necessary for an improved outcome.

## INTRODUCTION

Splenic marginal zone lymphoma (SMZL) is a rare, indolent subtype of non-Hodgkin lymphoma (NHL) that arises from B memory lymphocytes located in the marginal zone of secondary lymphoid follicles [1]. Most patients are asymptomatic, and the disease generally has a slow progression. The most common clinical sign is splenomegaly, which is found in 75% of cases. Approximately 25% of patients may experience anemia, thrombocytopenia, or leukocytosis [2]. Notably, approximately 20% of individuals with SMZL exhibit autoimmune manifestations [3]. While SMZL is known to be associated with various autoimmune disorders, the specific link between SMZL and chronic inflammatory demyelinating polyradiculoneuropathy (CIDP) has not been documented previously. CIDP is an acquired autoimmune condition affecting peripheral nerves and nerve roots. It usually presents with symmetrical weakness in both proximal and distal muscles, areflexia, and symptoms that progress for more than 8 weeks. SMZL is associated with immunodeficiency as a result of B-cell dysfunction, and the patient is at risk of developing heightened susceptibility to autoimmune manifestations, including CIDP. Chronic antigenic stimulation in lymphoproliferative disorders has been proposed as a contributing factor in the development of neuropathies [4]. While this study presents a single case, further research with larger sample sizes is required to establish a more comprehensive understanding of the interplay between CIDP and SMZL.

## CASE PRESENTATION

### Case history

We present the case of a 70-year-old male patient, a chronic shisha smoker with a known history of diabetes mellitus managed with insulin degludec and insulin aspart. His past surgical history included a laparoscopic cholecystectomy performed in 2018. The patient first presented to the gastrointestinal (GI) clinic on January 30, 2023, with complaints of left upper quadrant abdominal pain unrelated to food intake, accompanied by nighttime pruritus, night sweats, and the recent onset of diarrhea and steatorrhea. Physical examination revealed a soft abdomen with palpable splenomegaly, but no hepatomegaly, lymph node enlargement, or abdominal bruit. An external ultrasound showed splenomegaly, normal liver findings, and an enlarged prostate. Laboratory investigations (Table 1) revealed microcytic anemia (hemoglobin: 12.7 g/dL), leukopenia (white blood cell count: 3.8 × 10^9^/L), and a positive stool occult blood test. Liver and renal function tests remained within normal limits, as well as tumor markers, including alpha-fetoprotein (AFP), carbohydrate antigen 19-9 (CA 19-9), carcinoembryonic antigen (CEA), and prostate-specific antigen (PSA).

**Table 1:** Summary of laboratory findings in the patient at initial presentation

Test	Result	Units	Reference Range
**Complete Blood Count (CBC)**			
WBC	3.81	[x10^9/L]	4 – 10
RBC	4.67	[x10^12/L]	4.5 - 5.5
Hemoglobin (HGB)	12.70	[g/dL]	13 – 17
Hematocrit (HCT)	38.10	[%]	40 – 50
Mean Corpuscular Volume (MCV)	81.50	[fL]	83 – 99
Mean Corpuscular Hemoglobin (MCH)	27.20	[pg]	27 – 32
Mean Corpuscular Hemoglobin Conc. (MCHC)	33.40	[g/dL]	31.5 - 34.5
Red Cell Distribution Width (RDW)	16.00	[%]	11.5 - 14.5
Platelet Count (PLT)	122.00	[x10^9/L]	140 – 440
Mean Platelet Volume (MPV)	10.60	[fL]	7.2 - 12.0
**Chemistry Panel**			
Albumin, Serum	3.9	[g/dl]	3.2-4.6
Alkaline Phosphatase (ALK. PHOSPHATASE), Serum	166	[U/L]	50-116
Bilirubin (Direct), Serum	0.3	[mg/dl]	0-0.5
Bilirubin (Total), Serum	0.8	[mg/dl]	0.2-1.2
Creatinine (CRE2), Serum	1.04	[mg/dl]	0.72-1.25
Gamma-Glutamyl Transferase (GGT), Serum	66	[U/L]	12-64
SGOT (AST), Serum	17	[U/L]	5-34
SGPT (ALT), Serum	16	[U/L]	0-55
**Differential**			
Segmented neutrophil	66.30	[%]	-
Neutrophil absolute count	2.52	[x10^9/L]	2.0-7.0
Lymphocyte	25.60	[%]	-
Lymphocyte absolute count	0.98	[x10^9/L]	1.5-4.0
Monocyte	6.88	[%]	-
Monocyte absolute count	0.262	[x10^9/L]	0.1-0.8
Eosinophil	0.83	[%]	-
Eosinophil absolute count	0.03	[x10^9/L]	0.0-0.4
Basophil	0.38	[%]	-
Basophil absolute count	0.01	[x10^9/L]	0-0.2
**Other Tests**			
Occult blood in stool	Positive	-	-
Prothrombin Time (PT)	12.80	[seconds]	11.7-15.3
INR	0.93	-	-
Alpha-Fetoprotein (A.F.P) Serum	2.90	[ng/ml]	0-8

Magnetic resonance cholangiopancreatography (MRCP) revealed no evidence of hepatobiliary disease but confirmed massive splenomegaly (18.6 cm), raising suspicion for non-cirrhotic portal hypertension. He was scheduled for upper and lower endoscopy in February 2023 due to anemia and positive stool occult blood, to rule out malignancy.

**Figure 1:**
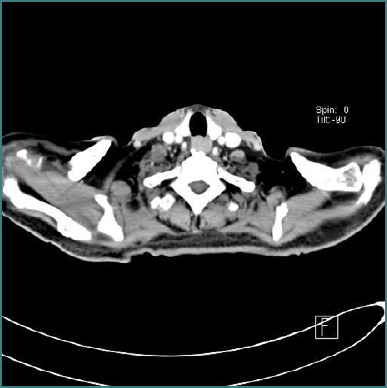
CT scan of the neck

**Figure 2:**
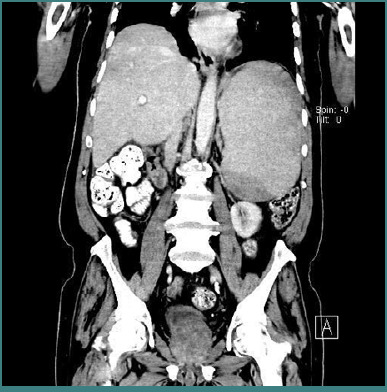
CT scan of the abdomen

In March 2023, endoscopy revealed gastroesophageal reflux disease (GERD) and tubulovillous adenomas with moderate dysplasia in cecal and rectal polyps, but no invasive malignancy. By April 2023, he reported worsening symptoms, including itching, weight loss, fatigue, and new symptoms of dizziness upon standing and mobilization. Despite his massive splenomegaly, his hepatobiliary system appeared unaffected on imaging, prompting further evaluation with CT of the neck, chest, abdomen, and pelvis. CT of the neck revealed supraventricular lymphadenopathy (Figure 1), and CT of the abdomen showed massive splenomegaly (Figure 2). By July 2023, the patient’s symptoms persisted, including weight loss, generalized fatigue, constant severe headaches, and decreased oral intake. He developed a change in sleep cycle and worsening dizziness. Repeat laboratory evaluation (Table 2) demonstrated pancytopenia, with a white blood cell count of 2.94 × 10^9^/L, red blood cell count of 3.59 × 10¹^2^/L, hemoglobin of 9.48 g/dL, and platelet count of 112 × 10^9^/L.

**Table 2:** Laboratory findings after treatment

Test	5/7/2023	13/7 after first dose	19/7 after second dose
WBC	2.94 [x10^9/L] (Low)	4.24 [x10^9/L] (Normal)	5.04 [x10^9/L] (Normal)
RBC	3.59 [x10^12/L] (Low)	3.42 [x10^12/L] (Low)	4.10 [x10^12/L] (Low)
HGB	9.48 [g/dL] (Low)	9.00 [g/dL] (Low)	10.80 [g/dL] (Low)
HCT	28.3 [%] (Low)	27.80 [%] (Low)	33.50 [%] (Low)
MCV	78.9 [fL] (Low)	81.40 [fL] (Low)	81.70 [fL] (Low)
MCH	26.4 [pg] (Normal)	26.30 [pg] (Normal)	26.40 [pg] (Normal)
MCHC	33.5 [g/dL] (Normal)	32.40 [g/dL] (Normal)	32.30 [g/dL] (Normal)
RDW	15.0 [%] (High)	17.20 [%] (High)	17.20 [%] (High)
PLT	112.0 [x10^9/L] (Low)	119.0 [x10^9/L] (Low)	139.0 [x10^9/L] (Low)
MPV	10.1 [fL] (Normal)	8.94 [fL] (Normal)	9.67 [fL] (Normal)
Segm. neutrophil	52.8 [%]	84.00 [%]	78.10 [%]
Neutrophil absolute	1.55 [x10^9/L] (Low)	3.56 [x10^9/L] (Normal)	3.94 [x10^9/L] (Normal)
Lymphocyte	31.9 [%]	8.40 [%]	15.80 [%]
Lymphocyte absolute	0.938 [x10^9/L] (Low)	0.36 [x10^9/L] (Low)	0.80 [x10^9/L] (Low)
Monocyte	11.2 [%]	4.25 [%]	4.32 [%]
Monocyte absolute	0.329 [x10^9/L] (Normal)	0.180 [x10^9/L] (Normal)	0.218 [x10^9/L] (Normal)
Eosinophil	0.148 [%]	0.10 [%]	0.23 [%]
Eosinophil absolute	0.004 [x10^9/L] (Normal)	0.00 [x10^9/L] (Normal)	0.01 [x10^9/L] (Normal)
Basophil	0.593 [%]	0.07 [%]	0.23 [%]
Basophil absolute	0.017 [x10^9/L] (Normal)	0.00 [x10^9/L] (Normal)	0.01 [x10^9/L] (Normal)

On July 5, 2023, a hematology evaluation was conducted, revealing proximal lower limb weakness but intact reflexes and sensation. There was a suspicion of malignancy, potentially indolent lymphoma, with a paraneoplastic syndrome such as CIDP.

The differential diagnosis included chronic lymphocytic leukemia (CLL), hairy cell leukemia (HCL), splenic marginal zone lymphoma (SMZL), Waldenström macroglobulinemia (WMG), idiopathic myelofibrosis (IMF), and systemic amyloidosis.

### Diagnostic approach

A comprehensive diagnostic workup was undertaken, including bone marrow biopsy, immunophenotyping, advanced imaging, and neurophysiological studies. Bone marrow examination revealed hypocellularity with increased lymphocytic infiltration, accounting for approximately 24% of viable cells. Immunophenotyping showed a B-cell predominance (56%) characterized by CD19+ and CD20+ expression, along with lambda light chain restriction. The neoplastic cells were negative for CD5, CD10, and CD103, findings consistent with a diagnosis of SMZL. Cross-sectional imaging (CT/MRI) confirmed massive splenomegaly, supraclavicular lymphadenopathy, portal hypertension, and pleural changes. Electromyography (EMG) indicated axonal sensory motor polyneuropathy, predominantly affecting the lower limbs.

### Patient management and outcomes

Following a comprehensive evaluation, including clinical assessments, laboratory tests, imaging studies, and nerve conduction studies, the patient was diagnosed with SMZL complicated by CIDP. Empirical treatment began with prednisolone at 1 mg/kg to address worsening neurological symptoms, including weakness and ataxia. This approach led to marked improvement in the patient's strength and neurological function. Rituximab therapy was initiated, with the patient receiving four cycles over four weeks.

After two cycles of rituximab, the patient experienced significant improvement in their clinical symptoms, including reduced fatigue, improved gait, and cessation of dizziness. Complete blood count (CBC) monitoring demonstrated a favorable hematologic response. By July 19, following the second rituximab cycle, the patient’s white blood cell count had increased to 5.04 × 10^9^/L, red blood cell count to 4.10 × 10¹^2^/L, and platelet count to 139 × 10^9^/L, indicating a favorable hematological response (Table 2).

## DISCUSSION

This case highlights the unusual association between CIDP and SMZL, outlining the challenges in diagnosing and managing patients with combined neurological and hematological conditions. CIDP is a chronic autoimmune neurological condition that presents with progressive weakness and loss of sensation due to the demyelination of peripheral nerves [5]. This is a rare non-Hodgkin's B-cell lymphoma, mainly involving the spleen and bone marrow, which presents as pancytopenia, splenomegaly, and constitutional symptoms [6]. In this patient, the most probable underlying cause for the CIDP was the SMZL. While most cases of CIDP are idiopathic, paraneoplastic forms associated with hematological malignancies, particularly lymphomas, have been reported [7]. Although they are likely to be complex, the exact mechanisms linking SMZL and CIDP remain unknown. It is thus conceivable that the immune dysregulation associated with lymphoma will trigger an autoimmune response against peripheral nerves leading to demyelination. Like other lymphomas, SMZL has been known to induce an abnormal immune response, which may result in the production of autoantibodies or immune cells attacking myelin sheaths, ultimately leading to CIDP. Alternatively, factors such as cytokines produced by the lymphoma could be 'driving' the immunological destruction of peripheral nerves [8]. Although the initial presentation of splenomegaly and left upper quadrant abdominal pain suggested a gastrointestinal or hematologic pathology—prompting the initial diagnostic workup—subsequent investigations ultimately led to the diagnosis of SMZL. Pancytopenia, coupled with proximalmuscle weakness and heavy perspiration later on, pointed toward a systemic disease process. The neuromuscular involvement, especially, deserved an extensive work-up, which included nerve conduction studies and thus confirmed the diagnosis of CIDP. Paraneoplastic syndromes should be considered when neurological symptoms cannot be accounted for by other means in patients with a known hematologic malignancy [9]. The early signs and symptoms of lymphoma may dominate the clinical presentation, potentially masking the diagnosis of CIDP secondary to SMZL. This bone marrow infiltration, resulting in pancytopenia, is not only an uncommon feature of SMZL but may also further complicate the management of CIDP due to its limited therapeutic options, including immunosuppressive treatment [10].

Although a well-recognized association exists between CIDP and hematologic malignancies, particularly B-cell lymphomas, the association of CIDP with SMZL is extremely rare [11]. The literature on this relation is very scant. One of the reasons for the low prevalence could be underdiagnosis, as sometimes symptoms of lymphoma mask its symptoms, or it may just point to some real biological rarity. The shared immunologic abnormalities suggest that CIDP may herald an underlying B-cell lymphoproliferative disorder, particularly marginal zone lymphomas. Malignant B-cells in SMZL may produce autoantibodies that cross-react with brain antigens or further induce immune responses against the myelin sheath, thereby triggering the autoimmune mechanisms of CIDP. Since the pathophysiological relationship between CIDP and SMZL is not fully understood, further research is needed. CIDP and SMZL patients can be challenging to manage [12].

There may be, for each condition, both conflicting and overlapping treatment approaches. Immunomodulatory treatments for CIDP which block the autoimmune attack on peripheral nerves include intravenous immunoglobulin (IVIG), corticosteroids, and plasmapheresis [13]. These therapies might, however, be less successful in the case of a paraneoplastic syndrome in which the underlying cancer triggers the autoimmune process. The treatments most commonly used for SMZL include splenectomy or systemic therapies, such as rituximab, an anti-CD20 monoclonal antibody that has been used with great success in the treatment of B-cell malignancies [14]. Interestingly, rituximab may offer therapeutic benefit in CIDP, particularly in cases associated with underlying B-cell dysregulation. This suggests that patients with concurrent SMZL and CIDP may experience significant clinical improvement with rituximab-based therapy. The cause of immune dysregulation in this patient, namely SMZL, treated with immunochemotherapy or targeted therapy, would likely reduce the symptoms of CIDP. Indeed, in patients where SMZL underlies the development of CIDP, treatment of the lymphoma itself may improve neurological symptoms. However, careful observation is required since CIDP may show an erratic course and lymphoma treatment is not always relieving the symptoms right away. Further, patients with pancytopenia, as in our case, are to be treated with caution in order to avoid hematologic deficits worsened by high-dose immunosuppressive therapies [15]. The rare association of CIDP with SMZL highlights the importance of a multidisciplinary approach in both diagnosis and treatment. Patients with hematological malignancies and unexplained neurological symptoms can present with para-neoplastic syndromes that clinicians must be aware of [16]. It is noteworthy that both conditions may improve with early treatment of the underlying cancer; therefore, establishing this diagnosis as early as possible is crucial. Further research into the pathophysiology underlying the comorbidity of CIDP and SMZL is necessary to determine the optimal treatment strategy for these patients.

## CONCLUSION

This case highlights the unusual but important association between chronic inflammatory demyelinating polyneuropathy and splenic marginal zone lymphoma. For many years, CIDP was considered a pure neurological disorder. Coincidences with hematological malignancies, such as in this case, require a high level of suspicion by the clinician in the presence of neurological deficits with systemic symptoms of splenomegaly and cytopenia. Both entities must be diagnosed early and managed through a multidisciplinary approach, as early immunosuppressive treatment can provide symptomatic relief in CIDP. In most instances, the indolent nature of SMZL allows for a conservative approach to treatment. This case also highlights the application of personalized treatment strategies and regular follow-up to monitor the course of both conditions, leading to better patient outcomes.
